# Supplementary material for: Multimodal emotion recognition via adaptive high-order transforme network
Source: PLoS One. 2025 Oct 27;20(10):e0333674. doi: 10.1371/journal.pone.0333674 (PMC12558493; doi:10.1371/journal.pone.0333674)
Supplement: Attachment — Submitted filename: Response to Reviewers.doc [file pone.0333674.s002.doc]

Multimodal Emotion Recognition via Adaptive High-order

Transformer Network

PLOS ONE

Sep 6 2025

**General comments to the reviewers and editor**

First of all, thank you very much for review our paper. We carefully considered your comments. Here, we explain how we revised the paper based on those comments and suggestions. We want to extend our appreciation for taking the time and effort necessary to provide such insightful guidance.

The revision, based on the review team’s collective input, includes a number of positive changes, all changes made to the text are in red color in the paper. Based on your helpful guidance, we mainly make the following changes:

- Analysis the effectiveness of proposed model
- Analysis and clarify the overall architecture and the detail of proposed block clearly.
- Check all the figures and equations
- Grammar and vocabulary mistakes are corrected
- Spelling errors and typos are scanned and corrected thoroughly

**1** Responses to Reviewer 1’s comments:

Reviewer 1 Comment 1

In Figure 1, Xv, XL, and XA respectively represent the inputs of video, text, and audio? It would be best if there had a brief description to facilitate reader’ reading. What is the abbreviation of BN? Is BN the BiLSTM Network?

**Response**

Thank you very much for your comments about our paper. X_v_, X_L_, and X_A_ respectively represent the inputs of video, text, and audio. We provide a detailed input description in Figure 1. "BN" denotes the Batch Normalization Layer, which is also annotated accordingly in Figure 1, as illustrated therein.

**Figure. 1. The architecture of adaptive high-order transformer network**

Reviewer 1 Comment 2

What are the benefits of using Conv1D in the Adaptive High-order Transformer Network designed in this paper? Especially when compared with the multi-scale convolution network commonly used in current image feature extraction.

**Response**

Thank you very much for your comments about our paper．For multimodal time-series inputs such as video and audio, using 1D convolution for feature learning offers distinct advantages over multi-scale convolution. Firstly, 1D convolution is computationally more efficient, as it operates solely along the temporal dimension, resulting in significantly fewer parameters and lower computational costs compared to 2D/3D multi-scale convolutions that process both spatial and temporal dimensions. This makes it particularly suitable for long sequences such as audio waveforms or video frame streams. Secondly, 1D convolution captures temporal dependencies more directly by adjusting the kernel size to flexibly extract local patterns of varying lengths—such as phonemes in audio or short-term actions in video—without the redundancy introduced by the complex branching structures typical in multi-scale convolution architectures. Furthermore, networks based on 1D convolution are lightweight, facilitate the stacking of deep layers for enhanced representational capacity, and integrate more seamlessly with recurrent neural networks or attention mechanisms, simplifying the construction of end-to-end temporal modeling pipelines. Although multi-scale convolution can capture features at multiple scales simultaneously, 1D convolution achieves efficient temporal modeling at a lower computational cost, making it more practical in scenarios with limited resources.

Reviewer 1 Comment 3

In the experimental comparison section, it is necessary to briefly explain why only the accuracy rate and the F1 score were selected for the comparison and analysis, because there are other index parameters for the evaluation of deep learning algorithms. For examples，the precision rate, the specificity and the ROC curve. Similarly, in the ablation experiment and parameter analysis sections, it is necessary to explain why only the F1 score was selected for the comparison and analysis.

**Response**

Thank you very much for your comments about our paper. In our multimodal emotion recognition experiments, we selected Accuracy and the F1 score as the core performance metrics for model comparison, primarily due to the prevalent issue of class imbalance inherent in such tasks. Accuracy offers an intuitive measure of the model’s overall prediction correctness, allowing readers to quickly grasp its general performance. However, in emotion datasets, certain categories (e.g., “happy” or “angry”) often significantly outnumber others (e.g., “sadness” or “surprise”). Relying solely on Accuracy may mask poor performance on minority classes, as results can be dominated by the majority classes. Therefore, we employ the F1 score—the harmonic mean of Precision and Recall—as a more central metric for evaluation. The F1 score provides a balanced assessment of the model’s ability to recognize each emotion category, with particular emphasis on its performance on infrequent classes. This is essential for ensuring a comprehensive and fair evaluation of an emotion recognition system.

In comparison, Precision alone may fail to reveal issues related to low Recall, while Specificity is more suited to binary negative case identification and loses interpretability in multi-class settings. Although the ROC curve offers a threshold-independent overview of model performance, its macro-level perspective does not directly reflect performance on specific minority classes, and it is less conducive to quick and straightforward numerical comparisons among multiple models. Moreover, since most related studies also use Accuracy and F1 score as standard quantitative metrics, our choice ensures consistency and facilitates fair comparison.

In the ablation studies and parameter analysis, we further narrow our evaluation focus to the F1 score—specifically the Macro-F1 score—for three main reasons: 1) The goal of ablation testing is to examine how each component contributes to the balanced performance of the model. The class-agnostic nature of Macro-F1 helps clearly indicate whether removing a module or altering parameters causes the model to bias toward majority classes. 2) Compared to Accuracy, the F1 score is more sensitive to class imbalance and variations in data distribution. It amplifies performance differences under various experimental settings, thereby supporting more nuanced and discriminative analysis. 3) Using one consistent and representative metric helps streamline results and avoids cluttered conclusions, making the analysis more focused and persuasive.

Again, we appreciate all your insightful comments. We worked hard to be responsive to them. Thank you for taking the time and energy to help us improve the paper.

Thank you and best regards
 yours sincerely

**2 Responses to Reviewer 2**’s comments:

Reviewer 2 General Comment

The paper introduces a combination of adaptive attention head/layer selection and high-order sparse feature learning for multimodal emotion recognition. The proposed method is evaluated on IEMOCAP and CMU-MOSEI datasets, achieving competitive performance. The work is clearly structured and includes comparisons and ablation studies. However, there are still issues related to terminology consistency, result presentation, reproducibility details, and efficiency evidence. These are mostly minor and can be resolved with targeted revisions.

**Response**

Thank you very much for your comments about our paper. We carefully considered your comments, all changes made to the text are in red color in the paper. In the following parts, you will find our responses to each of your points and suggestions. We are grateful for the time and energy you expended on our behalf.

Reviewer 2 Comment 1

The term “Cross-Modal Feature Fusion” is abbreviated inconsistently: the abstract uses “MCFF,” while the method section and figure captions use “CMFF.” Please unify the abbreviation throughout the paper and provide a table of abbreviations when they first appear. In the ablation study, “ATS” is used, but it should be “AST” (Adaptive Selection Transformer). Please unify this throughout the text and figure captions. In Figure 1, “Corss-Modal” should be corrected to “Cross-Modal.” Please check for consistent spelling across the paper. Equation (6) uses “MHS” but this should be “MHSA” (multi-head self-attention). Also, the temperature τ used in the selection mechanism is introduced but its range and default setting are missing. Suggestion: Add a short subsection “Notation & Abbreviations” at the beginning of the Method section with a clear abbreviation table. Correct figure captions and unify terminology. Provide a symbol table and default hyperparameters in the Appendix.

**Response**

Thank you very much for your comments about our paper. We check this description carefully,

We have updated the acronym "AHF" to "AHOT" throughout the entire manuscript. We have modified Equation (6) as follows:

| $\mathbf{Z}_{l}^{'}=\mathbf{E}_{l,0}\cdot MHSA\left(\mathbf{Z}_{l}\right)+\mathbf{Z}_{l}$, | (6) |
| --- | --- |

Temperature $\tau$ in AST is set to 5, the number of atoms and the low-rank matrix dimension in HOFL are set to 5 and 4096 respectively in our paper. The parameter settings are described in the "Datasets and Experimental Settings" section.

We have focused on revising Figures 1 and 2, while also unifying the abbreviations throughout the entire paper, including those in figures and tables. The details are as follows:

**Figure. 1. The architecture of adaptive high-order transformer network**

**Figure 2. Ablation study.**

We have added a glossary of abbreviations and a hyperparameter tables as shown below. Due to the formatting requirements of the journal template, these tables could not be inserted in their standard tabular form within the main text. However, all corresponding abbreviations and hyperparameters have been explicitly introduced and described in the respective sections. Additional experimental parameter settings are also described in the final paragraph of the "Datasets and Experimental Settings" section.

**Table 1. Abbreviations.**

| **Full Name** | **Abbreviation** |
| --- | --- |
| Adaptive High-order Transformer Network | AHOT |
| Adaptive Selection Transformer block | AST |
| Cross-modal Feature Fusion block | CMFF |
| Convolutional Neural Networks | CNNs |
| Residual Networks | ResNets |
| Long Short-Term Memory | LSTM |
| Sparse Interactive Attention Network | SIA-Net |
| Two-Phase Multi-Task Sentiment Analysis | TPMSA |
| Multimodal Embedding | ME |
| Mutual Transformer | MT |
| Deep Emotional Arousal Network | DEAN |
| High-Order Feature Learning module | HOFL |
| FeedForward Network | FFN |
| Multi-Head Self-Attention | MHSA |
| Principal Component Analysis | PCA |

**Table 2** Hyperparameter**.**

| **Hyperparameter** | **Value** |
| --- | --- |
| $\tau$ | 5 |
| $b$ | 5 |
| $v$ | 4096 |

Reviewer 2 Comment 2

On IEMOCAP, Table 1 shows category-wise accuracy (e.g., Neutral accuracy 72%, F1 71.5%), but the text discussing Figure 5 states the four-class accuracies are 88/86/87/87%. Please clarify whether these come from different splits, thresholds, or metrics, and unify the reporting. In Figure 6, the caption refers to “CMU-MOSI,” while elsewhere the dataset is called “CMU-MOSEI.” Please unify the dataset name. In Table 1, the column for Graph-MFN contains “–” for all entries. Please either provide the results for this baseline or explain in a footnote why they are unavailable/unreported.

**Response**

Thank you very much for your comments about our paper. The recognition accuracy for each category in Table 1 is as follows: Neutral reaches 72.3%, Angry 87%, Sad 85.9%, and Happy 88.3%. These results are consistent with the values shown on the main diagonal of the confusion matrix for the IEMOCAP dataset in Figure 5.

In multimodal emotion recognition, the confusion matrix offers an intuitive visualization of the model’s classification performance. The main diagonal entries represent the true positives—i.e., the number of samples correctly predicted for each emotion category—reflecting the overall accuracy of the model. The off-diagonal elements, on the other hand, reveal misclassification patterns (such as “Happy” being incorrectly predicted as “Sad”), which help identify specific confusion between categories.

Moreover, we have revised the caption of Figure 6 to: t-SNE visualization on the CMU-MOSEI dataset. P, WP, HP, WN, N, HN, and NT represent Positive, Weakly Positive, Highly Positive, Weakly Negative, Negative, Highly Negative, and Neutral, respectively.

The original Graph-MFN paper conducted experiments solely on the CMU-MOSEI dataset and did not include specific experiments on the IEMOCAP dataset. Moreover, the authors did not release the official code or model, making it difficult to reproduce their reported results accurately through self-implementation. To ensure fairness and completeness in the comparative experiments across both datasets, we have marked the results of Graph-MFN on the IEMOCAP dataset as “not available”. The original Graph-MFN paper can be referenced as follows:

Wu, J.F.; Mai, S.J.; Hu, H.F. Graph capsule aggregation for unaligned multimodal sequences. In Proceedings of the 2021 International Conference on Multimodal Interaction, Montreal, Canada, 18–22 October 2021.

Additionally, we have added the footnote "* The best results are highlighted in bold, -denotes that the experiment is not conducted.".

Reviewer 2 Comment 3

The paper claims that adaptive head/layer selection reduces redundancy and improves efficiency, but no evidence is provided in terms of FLOPs, latency, parameter counts, or energy. At least on CMU-MOSEI, please report training/inference time, parameter counts, FLOPs, and accuracy-efficiency trade-offs compared with the non-adaptive version.

**Response**

Thank you very much for your comments about our paper. Based on your suggestions, we have incorporated an analysis of model effectiveness, as detailed below.

**Table 3. The analysis of model** **efficiency.**

| **Method** | **Head** | **FLOPs(G)** | **Parameter (M)** | **Total Inference Time(s)** | **Acc_2_(%)** |
| --- | --- | --- | --- | --- | --- |
| T2T-ViT[51] | 7 | 8.5 | 12 | 48.24 | 83.0 |
| AST | 4 | 3.9 | 5.5 | 21.92 | 82.7 |
| AST | 5 | 4.3 | 6.8 | 24.89 | 82.8 |

Table 3 presents the analysis of model efficiency. We adopt the T2T-ViT model as the backbone, which utilizes full self-attention heads and transformer blocks. The AST (Adaptive Sparse Transformer) mechanism proposed in this work effectively controls the number of attention heads. In our emotion recognition experiments, the number of heads was constrained to approximately 4 and 5. Experimental results show that, compared to the T2T-ViT model with 7 heads, AST achieves good recognition performance even with fewer parameters. Additionally, while the performance of models with 4 and 5 heads is nearly identical—with Head=5 yielding only a marginal improvement of 0.1%—the model with 4 heads is selected as the optimal configuration due to its lower parameter count and computational cost. In conclusion, the results demonstrate the effectiveness of the AST mechanism in reducing model redundancy. Moreover, AST demonstrates a significant advantage in terms of the accuracy-efficiency trade-off.

**Reference**

[51] Yuan L, Chen Y, Wang T, Yu W, Shi Y, Jiang Z H, Tay F E H, Feng J, Yan S. Tokens-to-token vit: Training vision transformers from scratch on imagenet. In Proceedings of the IEEE/CVF International Conference on Computer Vision, Montreal, QC, Canada, 10-17 October 2021.

Reviewer 2 Comment 4

Method names: the abstract uses “adaptive head selection Transformer blocks (AST) and cross-modal feature fusion blocks (MCFF),” while the Method section titles use slightly different forms. Please unify capitalization and plurality.

References: items #15 and #41 are duplicates (“Deep emotional arousal network,” Information Fusion 2022). Please remove duplicates and recheck numbering.

**Response**

Thank you very much for your comments about our paper. Based on your comments, we have updated the corresponding section in the abstract as follows: Adaptive High-order Transformer Network (AHOT), Adaptive Selection Transformer block (AST) and Cross-modal Feature Fusion block (CMFF). We have updated the acronym "AHF" to "AHOT" throughout the entire manuscript. This ensures full consistency with the Methods section below. In accordance with the journal’s formatting guidelines regarding section headings, initial capitalization has not been applied here in order to comply with the specific style requirements.

We have removed Reference 15 and replaced Reference 6 with a recent publication in the field of multimodal emotion recognition. All references have been renumbered accordingly and carefully verified. The specific changes are as follows:

**Reference**

[6] Liu S, Gao P, Li Y, Fu W, Ding W. Multi-modal fusion network with complementarity and importance for emotion recognition. *Inf. Fusion* **2023**, 619, 679-694.

[15] Liu S, Huang S, Fu W, Liu JCW. A descriptive human visual cognitive strategy using graph neural network for facial expression recognition. *Int J Mach Learn Cyb*, **2024**, 15, 19-35.

Again, we appreciate all your insightful comments. We worked hard to be responsive to them. Thank you for taking the time and energy to help us improve the paper.

Thank you and best regards
 yours sincerely
